# Right Atrial Phasic Function in Heart Failure with Preserved Ejection Fraction: Cardiac Magnetic Resonance Feature Tracking and Outcomes

**DOI:** 10.3390/jcm12165179

**Published:** 2023-08-09

**Authors:** Robert Schönbauer, Fiona Hana, Franz Duca, Matthias Koschutnik, Carolina Donà, Christian Nitsche, Michael Sponder, Max Lenz, Jonghui Lee, Christian Loewe, Christian Hengstenberg, Julia Mascherbauer, Andreas Kammerlander

**Affiliations:** 1 Department of Internal Medicine II, Division of Cardiology, Medical University of Vienna, 1090 Vienna, Austria; robert.schoenbauer@meduniwien.ac.at (R.S.); fiona.hana@meduniwien.ac.at (F.H.); franz.duca@meduniwien.ac.at (F.D.); matthias.koschutnik@meduniwien.ac.at (M.K.); carolina.dona@meduniwien.ac.at (C.D.); christian.nitsche@meduniwien.ac.at (C.N.); michael.sponder@meduniwien.ac.at (M.S.); max.lenz@meduniwien.ac.at (M.L.); jonghui.lee@meduniwien.ac.at (J.L.); christian.hengstenberg@meduniwien.ac.at (C.H.);; 2 Department of Bioimiging and Image-Guided Therapy, Division of Cardiovascular and Interventional Radiology, Medical University of Vienna, 1090 Vienna, Austria; christian.loewe@meduniwien.ac.at

**Keywords:** HFpEF, atrial fibrillation, atrial function, CMR

## Abstract

Background: This study sought to investigate the prognostic impact of right atrial (RA) size and function in patients with heart failure with preserved ejection fraction (HFpEF) in sinus rhythm (SR) and atrial fibrillation (AF). Methods: Consecutive HFpEF patients were enrolled and indexed RA volumes and emptying fractions (RA-EF) were assessed by cardiac magnetic resonance imaging (CMR). For patients in SR, feature tracking of the RA wall was performed during CMR. In addition, all patients underwent right and left heart catheterization and 6 min walk distance (6MWD) and N-terminal prohormone of brain natriuretic peptide (NT-proBNP) evaluations. We prospectively followed patients and used Cox regression models to determine the association of RA size and function with a composite endpoint of heart failure hospitalization and cardiovascular death. Results: A total of 188 patients (71% female patients, 70 ± 8 years old) were included. Ninety-two patients (49%) were in persistent AF. Eighty-five patients reached the combined endpoint during a follow-up of 69 (42–97) months. After a multivariate cox regression analysis, the impaired RA reservoir strain (HR 0.949; 95% CI [0.909–0.990], *p* = 0.016), the RA reservoir strain rate (HR 0.991; 95% CI [0.983–0.999], *p* = 0.028), the RA conduit strain (HR 0.932; 95% CI [0.879–0.988], *p* = 0.019), and the RA conduit strain rate (HR 0.989; 95% CI [0.881–0.997], *p* = 0.011) were significantly associated with a worse outcome for patients in SR. In persistent AF, no RA imaging parameter was related to outcome after a multivariate regression analysis. Conclusions: In HFpEF patients in SR, CMR parameters of impaired RA conduit and reservoir function are associated with dismal cardiovascular outcomes. In persistent AF, RA parameters lose their prognostic ability.

## 1. Introduction

Heart failure is a pandemic, affecting millions of people worldwide. About 50% of heart failure patients suffer from heart failure with preserved ejection fraction (HFpEF) [1]. As the population ages and the prevalence of risk factors such as obesity and hypertension increases, HFpEF is expected to become an even more significant health burden. While HFpEF is associated with substantial morbidity and mortality, similar to heart failure with reduced ejection fraction (HFrEF), diagnosis of HFpEF can be challenging when compared to HFrEF, where diagnosis mainly relies on imaging of reduced systolic ejection fractions. Therefore, a better understanding of HFpEF is of particular interest [2]. Despite its high prevalence, the pathomechanisms of HFpEF still are not completely understood. In the “traditional” HFpEF model, left ventricular (LV) dysfunction leads to left atrial (LA) hypertension-related remodeling, which in turn further leads to pulmonary hypertension and right atrial (RA)-pressure-increase-associated remodeling [3]. In contrast to this, during recent years, an alternate HFpEF model has emerged, suggesting simultaneous global cardiac remodeling and dysfunction caused by systemic microvascular endothelial inflammation as an underlying mechanism for the development of HFpEF [3,4]. LA hypertrophy and microvascular dysfunction have already been shown in early HFpEF stages in an animal model, which further supports this emerging HFpEF model of global cardiac remodeling [5]. Impaired LA function is a major prognostic determinant in HFpEF [6,7]. In particular, the LA conduit function plays a major role regarding exercise capacity and cardiovascular outcome [8]. Additionally, impaired right ventricular performance and pulmonary artery hypertension are well-known predictors for a dismal outcome in HFpEF [9,10]. In this context, it is of interest whether RA geometry and function also have any influence on the outcome, as until now data regarding the impact of RA function on exercise capacity, outcome and pulmonary vascular performance in patients with HFpEF are sparse [11,12]. Furthermore, up to 65% of the HFpEF population suffer from atrial fibrillation (AF) [13].

Therefore, we conducted this trial to evaluate:

(1) The impact of phasic RA volumes und function on cardiovascular outcomes for HFpEF patients in SR;
(2) The correlation of phasic RA volumes and function with markers of cardiovascular and pulmonary vascular performance;
(3) The impact of RA volumes and ejection fraction on cardiovascular outcome for HFpEF patients in persistent AF;
(4) The correlation of RA volumes and ejection fraction with markers of cardiovascular and pulmonary vascular performance.

RA phasic volume and wall deformation analyses were performed using standard cardiac magnetic resonance (CMR)-balanced steady-state free precision (bSSFP) cine sequences, as CMR enables excellent imaging of the atrial wall in comparison to standard echocardiography [11,14,15]. In addition, RA wall deformation analysis was performed only for patients in SR using dedicated software, as wall deformation analysis cannot be reliably performed during AF.

## 2. Materials and Methods

### 2.1. Study Design

This prospective observational study was executed at the Medical University of Vienna and approved by the local ethical committee (EK No. 796/2010). Between December 2010 and March 2017 consecutive patients were screened at the HFpEF outpatient clinic for participation in the study. All enrolled patients gave written informed consent. All methods were performed in accordance with relevant guidelines and regulations.

### 2.2. Clinical Definitions

HFpEF diagnosis was established according to the recommendations of the European Society of Cardiology [16]: (1) signs and/or symptoms of heart failure; (2) LV EF ≥ 50%; (3) N-terminal prohormone of brain natriuretic peptide (NT-proBNP) > 220 pg/mL; (4) markers for diastolic dysfunction and/or myocardiac structure changes like LA enlargement and LV hypertrophy.

In addition, the diagnosis had to be invasively confirmed by elevated pulmonary artery wedge pressure in right heart catheterization. Reasons for exclusion were hemodynamically relevant coronary artery disease, live expectancy <1 year, hemodynamically relevant valve disease, relevant structural heart disease like congenital heart disease, and myocardial storage disease including cardiac amyloidosis, which was specifically screened for [17].

### 2.3. Outcome Measures

Follow-up consisted of ambulatory visits every 6 months and telephone calls. The primary combined study endpoint included hospitalization for heart failure and death by cardiovascular causes. Endpoints were detected by follow-up visits and telephone calls. Ascertained endpoints were then confirmed by the study’s adjudication committee, which was blinded to the patient´s baseline characteristics as well as imaging and hemodynamic data.

### 2.4. Assessment Techniques

#### 2.4.1. Exercise Capacity

The individual submaximal exercise capacity of all study participants was evaluated by the 6 min walking distance (6MWD) on a 50 m indoor track [18].

#### 2.4.2. Transthoracic Echocardiography

All enrolled patients underwent transthoracic echocardiography executed by board certified physicians on commercially available scanners, such as Vivid S70 (GE Healthcare, Chicago, IL, USA). LV EF was evaluated by the biplane Simpson’s technique. Transmitral flow was assessed by the use of a pulsed wave Doppler. Septal and lateral mitral annular wall motion was assessed by the use of a pulsed wave tissue Doppler. E/e was then further calculated.

#### 2.4.3. Invasive Hemodynamic Assessment

Right heart catheterization was performed via femoral or jugular venous access. Pressures were documented as the mean of at least eight consecutive heart cycles using CathCorLX (Siemens AG, Berlin and Munich, Germany). The following pressures were documented: mean pulmonary artery wedge pressure, pulmonary artery systolic and diastolic pressure and mean pulmonary artery pressure. The LV end-diastolic pressure was manually checked, and the cardiac output was measured by thermodilution. Derived hemodynamic parameters were calculated with standard formulas. Hemodynamic assessment coronary angiography was subsequently carried out in the same procedure.

#### 2.4.4. Cardiac Magnetic Resonance Imaging

All study participants underwent CMR, which was performed on a 1.5 Tesla scanner (MAGNETOM Avanto fit, Siemens Healthcare GmbH, Erlangen, Germany). Patients with relevant renal insufficiency with an estimated glomerular filtration rate of <30 mL/min/1.73 m^2^ were excluded. CMR was performed according to standard protocols [19], including late gadolinium enhancement imaging (0.1 mmoL/kg gadobutrol, Gadovist, Bayer Vital GmbH, Leverkusen, Germany) and T1 mapping using the modified Look-Locker inversion (MOLLI) sequence [20]. For pre-contrast T1 mapping, electrocardiographically triggered MOLLI was applied using a 5(3)3 prototype (5 acquisition heartbeats followed by 3 recovery heartbeats and a further 3 acquisition heartbeats). For post-contrast T1 mapping, a 4(1)3(1)2 prototype was used. T1 values from a mid-cavity two- and four-chamber view were averaged. Regions of interest for T1 blood pool values were derived from sufficient distance to papillary muscles and the endomyocardial border and MOLLI extracellular volume (ECV) were then calculated [7].

##### Right Atrial Chamber Evaluation

**Figure 1:**
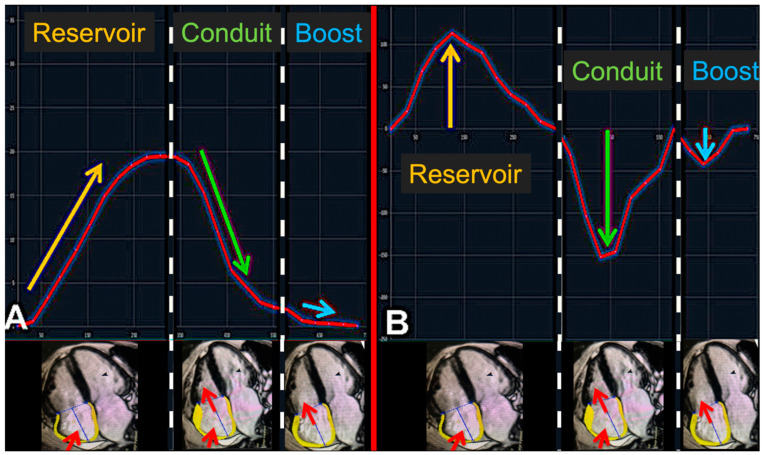
Right atrial phasic transport function visualized by CMR feature tracking. Panel (**A**): longitudinal strain. Panel B: longitudinal strain rate analysis. The red curve depicts the time-dependent trend of right atrial longitudinal dilation in % of baseline dilation. Panel (**B**): longitudinal strain rate analysis. The red curve depicts the time-dependent trend of velocity of longitudinal dilation in %/s of baseline dilation.

The phasic RA function is divided into three parts (Figure 1):

(1) Reservoir function: atrial diastole during ventricular systole.
(2) Conduit function: subsequent passive atrial emptying starts with the opening of the atrio-ventricular valves.
(3) Booster pump function: active atrial emptying (which is lost in AF).

The RA chamber was evaluated offline on a remote work station using commercially available software (cvi42, version 5, Circle Cardiovascular Imaging Inc., Calgary, AB, Canada) [11]. The RA volume was assessed in four-chamber view via the mono plane area length method. Three different RA volumes were calculated: (1) the largest volume directly before tricuspid valve opening; (2) the smallest volume directly before closing of the tricuspid valve; and (3) for patients in SR, the volume directly before the onset of the P-wave on electrocardiography, which marks the onset of the booster pump function. RA conduit EF, booster pump EF, and total EF were then further calculated.

For RA strain analyses, dedicated feature tracking software was used (Tissue Tracking V5 plugin, cmr42, Circle Cardiovascular Imaging Inc., Calgary, AB, Canada) [11,12]. RA endo- and epi-cardial borders were manually marked monoplane in a four-chamber view at the end of ventricular systole (directly before closing of the tricuspid valve). Then, RA wall motion tracking over the whole cardiac cycle was performed automatically and manual adjustments were made if needed. According to this wall motion analysis, corresponding strain and strain rate curves were drawn (Figure 1) and strain and strain rate-values of reservoir, conduit, and booster pump functions were calculated. This whole process was repeated three times and the average values were documented.

### 2.5. Statistical Analysis

Analyses were performed using dedicated software (IBM SPSS 28 SPSS Inc., Chicago, IL, USA). For all tests, the significance level was set to *p* < 0.05. Continuous variables were compared using the Mann–Whitney U test; for dichotomous variables, the chi-square test was used. A Spearman (*ρ*) test was used to find factors correlated with RA phasic function and volumetric analyses.

Continuous variables are expressed as means ± standard deviation and categorical variables as numbers and percentages. For definition of the factors associated with AF, a univariate logistic regression analysis was conducted for each baseline characteristic parameter.

To analyze possible associations with the combined endpoint of hospitalization for heart failure and cardiovascular death, a univariable cox regression analysis was performed for each RA volumetric and strain parameter in SR and AF. In addition, adjusted hazard ratios were performed for all significant RA parameters, which showed significant association in a univariable cox regression analysis adjusted for age, NT-proBNP, RV EF, and NYHA class.

A Kaplan–Meier plot was used to verify the time-dependent discriminative power of the RA conduit strain rate on cardiovascular outcome.

## 3. Results

### 3.1. Study Population

From December 2010 to November 2017, 343 HFpEF patients were screened in our HFpEF outpatient clinic. A total of fifty patients were excluded due to the following reasons: NT-proBNP < 220 pg/mL (n = 16), significant coronary artery disease (n = 18), chronic thromboembolic pulmonary hypertension (n = 1), and cardiac amyloidosis (n = 15). CMR was not performed in 98 patients due to contraindications (n = 46) or refusal (n = 52). Seven patients were excluded from our analysis due to insufficient CMR image quality.

One hundred and eighty-eight patients were included in the final study (71% female patients, 70 ± 8 years old). Table 1 displays the clinical, CMR, and invasive hemodynamic baseline characteristics according to the heart rhythm during CMR acquisition. Ninety-six patients (51%) were in SR and ninety-two (49%) in AF. All AF patients were in persistent AF. Thirty patients (31%) in SR at the time of CMR had a history of paroxysmal AF but did not differ from SR patients according to baseline characteristics. The proportion of female patients was inferior in the cohort of patients with persistent AF (78% vs. 63%; *p* = 0.026). Patients in AF were older (*p* = 0.023), had a higher heart rate (*p =* 0.010), and were in a lower New York Heart Association functional class (*p =* 0.003). Furthermore, they had higher serum levels of NT-proBNP and gamma-glutamyl-transferase (each *p* < 0.001, respectively).

According to CMR imaging parameters, patients in persistent AF had a marked pronounced bi-atrial and RV dilatation, as well as an impaired RV and LV ejection fraction and an elevated proportion of MOLLI-ECV (each *p* < 0.001, respectively) when compared to patients in SR.

In invasive hemodynamic measurements, persistent AF patients showed a decreased cardiac output (*p* = 0.028).

In comparison to recently published reference values of healthy subjects [21], reservoir, conduit, and booster pump strain and strain rate values of our HFpEF study population were reduced. When compared to SR, patients in AF had significant pronounced maximal and minimal RA volume index, as well as an inferior RA EF (each *p* < 0.001, respectively) (Table 2).

### 3.2. Right Atrial Function and Cardiovascular Outcome in Sinus Rhythm and Atrial Fibrillation

Patients were followed for 69 (42–97) months. The study endpoint was defined as a combination of hospitalization for heart failure and death due to cardiovascular causes. A total of 85 patients reached the combined endpoint, of which 18 were cardiovascular deaths. Table 3 summarizes the uni- and multi-variate findings regarding the association of RA volumetric and strain-related parameters to cardiovascular outcomes in SR and AF.

**Figure 2:**
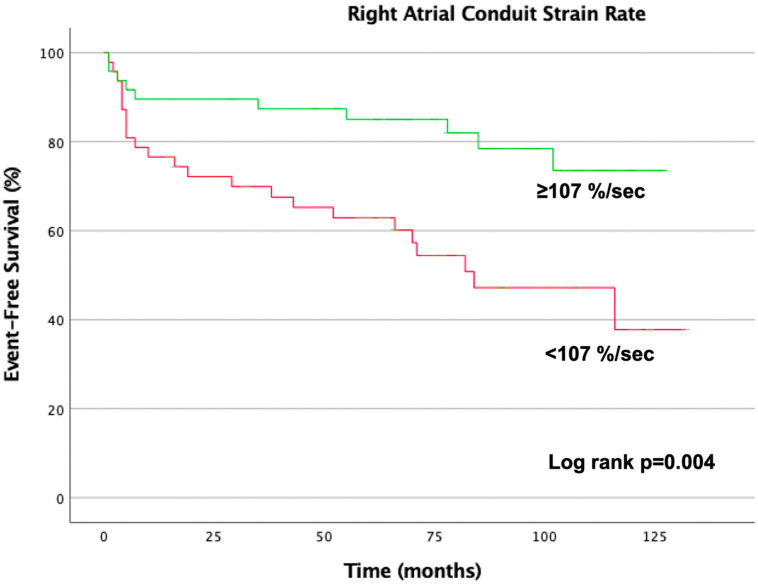
Kaplan–Meier plot according to right atrial conduit strain conduit function. Patients were stratified according to the median right atrial conduit strain rate (107−%/s). The green line indicates the event-free survival curve for patients with right atrial conduit strain rate ≥107−.

Thirty-four patients (35%) of the cohort in SR reached the combined endpoint. No RA volumetric parameter was related to the outcome. In contrast, the RA reservoir strain and strain rate, as well as the RA conduit strain and strain rate, were significantly related to the combined endpoint (Figure 2). After multivariate cox regression adjusted for age, NT-proBNP level, right ventricular ejection fraction, and HF functional class, the impaired RA strain (*p* = 0.016) and strain rate (*p* = 0.028), as well as the impaired RA conduit strain (*p* = 0.019) and strain rate (*p* = 0.011), were still significantly related to the cardiovascular outcome (Table 3).

Fifty-one patients (55%) of the patient cohort in AF reached the combined endpoint. In univariate cox regression, an impaired RA total EF was related to the combined endpoint (*p* = 0.026). However, this did not hold true after a multivariate regression analysis.

### 3.3. Right Atrial Function in Correlation to Functional Capacity, NT-proBNP Levels, and Pulmonary Vascular Function

Table 4 summarizes the univariate correlation of various RA static and dynamic volumetric and strain parameters to 6MWD, NT-proBNP levels, systolic pulmonary artery pressure (sPAP), and pulmonary vascular resistance (PVR) for patients in SR and AF.

**Figure 3:**
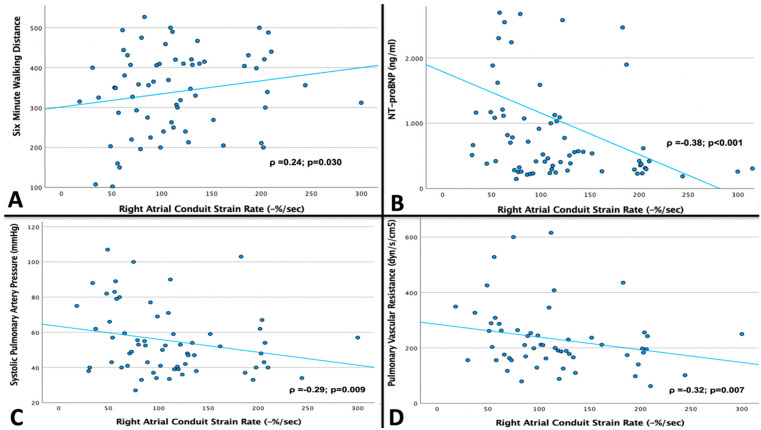
Univariate linear regression analysis displaying the significant correlation of right atrial conduit strain rate with the six-minute walking distance (panel (**A**)), NT-proBNP levels (panel (**B**)), systolic pulmonary artery pressure (panel (**C**)), and pulmonary vascular resistance (panel (**D**)).

For patients in SR, parameters of the RA booster pump function showed no correlation to any of the aforementioned parameters at all. In contrast, parameters of the RA conduit function showed a robust correlation to all of the mentioned parameters (Figure 3). Furthermore, parameters of the RA reservoir function were correlated to NT-proBNP levels, sPAP, and PVR. Impaired RA total EF showed a significant correlation with elevated NT-proBNP levels and increased PVR (*p* = 0.011 and *p* = 0.026, respectively). Increased end systolic, pre-A, and end diastolic RA volume indexes were also correlated with elevated NT-proBNP levels (*p* = 0.011, *p* = 0.009, and *p* = 0.002, respectively).

In contrast, for patients in persistent AF, no RA parameter was correlated to 6MWD, NT-proBNP levels, sPAP, or PVR.

## 4. Discussion

In this study combining data of a CMR feature tracking assessment of the RA function with invasive hemodynamics with clinical data and outcomes in HFpEF patients in persistent AF vs. SR, we report three main findings:

(1) For patients in SR, parameters of the RA reservoir and conduit function show (in contrast to booster pump function) a robust relation to cardiovascular outcome, even after adjustment for well-known risk factors in HFpEF.
(2) An impaired RA conduit function shows a significant association with an impaired exercise capacity (6MWD, NT-proBNP) and an impaired pulmonary vascular function (sPAP, PVR).
(3) Regarding baseline characteristics, patients with persistent AF showed markers of pronounced disease progression. However, parameters of RA function did not relate to cardiovascular outcome, nor did they correlate to parameters of exercise capacity and pulmonary vascular function in this patient population.

### 4.1. Prognostic Value of Right Atrial Volume and Function

Data regarding the prognostic impact of phasic RA function are very much limited. Regarding the prognostic value of RA function, our findings very much replicate the findings of Jain S. et al. [11]. They also showed a strong relation between an impaired RA reservoir and conduit function in the worsening of cardiovascular outcomes in a mixed study population of patients with HFpEF, systolic HF, and a control group without HF [11]. Interestingly, the RA volume index and RA booster pump function were not related to the outcome in their findings, as well as in ours. These findings are in contrast to two previous studies investigating the prognostic impact of the maximum RA volume index as assessed by echocardiography [22] and CMR [23] in a patient cohort suffering from chronic systolic HF, where an independent link to adverse outcomes was shown. In summary, these findings implicate that maximum RAVi could be more relevant as a prognostic risk factor in HFrEF rather than in HFpEF. However, due to still very limited data, further studies on the prognostic impact of RA phasic volumes and function both in HFpEF and HFrEF are warranted to replicate these findings and, moreover, find possible reasons for them.

### 4.2. Right Atrial Phasic Function and Its Relation with Exercise Capacity and Pulmonary Vascular Function

To the best of our knowledge, this is the first study correlating CMR feature tracking assessed parameters of phasic RA function with parameters of exercise capacity and invasive hemodynamics (Table 4 and Figure 3). Parameters of RA conduit and reservoir function showed a close correlation with NT-proBNP, sPAP, and PVR. Furthermore, an impaired RA conduit function was correlated with decreased 6MWD. These findings could at least partly be an explanation for the prognostic impact of RA conduit and reservoir function. On the other hand, a reduced RA conduit and reservoir function may reflect an impaired central venous capacitance, a central mechanism in patients suffering from HFpEF [24].

### 4.3. Prognostic Impact of Right Atrial Function in Heart Failure with Preserved Ejection Fraction and Atrial Fibrillation

About 50% of our patient population were in persistent atrial fibrillation, which reflects data from previous studies. As previously described, HFpEF patients in persistent AF represent a population with a more advanced stage of disease [25]. Additionally, in our study population, patients in persistent AF were older, in a higher NYHA functional class, and had higher levels of NT-proBNP. Regarding CMR imaging parameters, these patients had markedly pronounced RV end diastolic diameters, decreased RV EF, and elevated LV ECVs (Table 1). Nevertheless, in the study population of HFpEF patients with persistent AF, RA parameters did not show any prognostic impact regarding the cardiovascular outcome (Table 3), not even in a univariate analysis. AF and, in particular, persistent AF are well-established risk factors for adverse outcome in HFpEF [13]. Our data implicate that further risk stratification by evaluating RA function does not come with any further prognostic information for HFpEF patients in persistent AF.

## 5. Clinical Implications

Our data imply that imaging parameters of RA function can be used as an independent tool for risk stratification in HFpEF patients in SR. Accordingly, very recently, reference values for the RA strain of healthy subjects were published. On the other hand, repeated measurements of RA function can be used as a tool for disease progress observation in HFpEF or for monitoring positive or adverse effects of HFpEF-specific therapeutic agents, like angiotensin–neprilysin inhibitors [26] or sodium/glucose cotransporter 2 inhibitors [27,28].

Due to yet very limited data on the prognostic impact of parameters of RA function, further large-scale studies are still warranted.

## 6. Conclusions

For HFpEF patients in SR, impaired strain-related parameters of the RA reservoir and conduit function can serve as an independent predictor for dismal cardiovascular outcomes. Furthermore, the RA reservoir and conduit function exhibit a close correlation with exercise capacity and pulmonary vascular function. These findings implicate the use of RA functional parameters for HFpEF disease progression monitoring and for targeting HFpEF-specific therapeutic agents [26,27,28,29]. Nevertheless, due to still very limited data, large-scale studies are warranted on the clinical impact of RA volume and function in HFpEF.

## 7. Limitations

As this study was not multicentric, a center-specific bias cannot be completely ruled out. However, single center studies do also have some advantages, such as homogenous patient selection, continuous workflow, and constant follow up. As continuous electrocardiogram monitoring was not performed for every patient, asymptomatic AF episodes may have been missed and the real-life burden of AF episodes might be higher. The cross-sectional observational study design limits conclusions about cause–effect relationships.

## Figures and Tables

**Table 1:** Baseline characteristics.

Variable	Sinus Rhythm (n = 96)	AF (n = 92)	*p*-Value
**Clinical parameters**			
Age, y	69 ± 9	72 ± 8	**0.023**
Female sex, n (%)	75 (78)	58 (63)	**0.026**
Body mass index, kg/m^2^	31 ± 6	30 ± 6	0.432
Paroxysmal AF (%)	30 (31)	0 (0)	**<0.001**
Persistent AF (%)	0 (0)	92 (100)	**<0.001**
Diabetes mellitus type II, n (%)	37 (39)	30 (33)	0.446
Hyperlipidemia, n (%)	56 (58)	44 (48)	0.144
Arterial hypertension, n (%)	89 (93)	89 (97)	0.497
Heart rate, (beats/min)	69 ± 14	73 ± 13	**0.010**
6MWD, m	344 ± 112	318 ± 120	0.109
Sleep apnea, n (%)	8 (8)	12 (13)	0.350
COPD, n (%)	26 (27)	32 (35)	0.190
NYHA functional class, n (%)			**0.003**
II	45 (47)	26 (28)	
III	49 (51)	58 (63)	
IV	2 (2)	8 (9)	
NT-proBNP, ng/mL	0.52 (0.32 to 1.16)	1.60 (1.04 to 2.42)	**<0.001**
Glycated hemoglobin, %	6.1 ± 1.3	6.1 ± 0.7	0.470
eGFR, mL/min/1.73 m^2^	60 ± 18	62 ± 19	0.719
Gamma-Glutamyl-Transferase, U/L	27 (19 to 41)	47 (29 to 94)	**<0.001**
HMG-CoA reductase inhibitor, n (%)	48 (50)	36 (39)	0.106
Betablocker, n (%)	67 (70)	66 (72)	1.000
Diuretics, n (%)	69 (72)	72 (78)	0.605
ACE inhibitor, n (%)	21 (22)	31 (34)	0.104
AT II receptor antagonist, n (%)	41 (43)	33 (36)	0.296
**Cardiac magnetic resonance imaging parameters**			
LV end-diastolic diameter, mm	47 ± 6	47 ± 6	0.565
RV end-diastolic diameter, mm	39 ± 7	43 ± 8	**<0.001**
Interventricular septum, mm	11 ± 2	11 ± 2	0.754
LA diameter, mm	68 ± 8	75 ± 10	**<0.001**
LA area, cm^2^	30 ± 7	37 ± 9	**<0.001**
RA diameter, mm	61 ± 7	70 ± 10	**<0.001**
RA area, cm^2^	25 ± 5	35 ± 11	**<0.001**
LV ejection fraction, %	68 ± 9	61 ± 6	**<0.001**
LV end-diastolic volume, mL	129 ± 45	123 ± 42	0.793
Cardiac output, L/min	5.6 ± 1.9	5.1 ± 2.9	0.072
RV ejection fraction, %	56 ± 11	47 ± 10	**<0.001**
RV end-diastolic volume, mL	144 ± 46	162 ± 65	0.053
MOLLI-ECV	28.0 ± 3.2	31.2 ± 4.8	**<0.001**
**Invasive hemodynamics**			
Systolic PAP, mmHg	53 ± 20	54 ± 18	0.589
Diastolic PAP, mmHg	21 ± 8	23 ± 7	0.112
Mean PAP, mmHg	33 ± 11	35 ± 10	0.379
PAWP, mmHg	19 ± 6	20 ± 6	0.154
LV end-diastolic pressure, mmHg	21 ± 7	20 ± 6	0.439
TPG, mmHg	14 ± 7	14 ± 7	0.860
Diastolic pressure gradient, mmHg	2.0 (−1.0 to 5.0)	2.0 (−2.0 to 6.0)	0.634
CO thermodilution, L/min	5.5 ± 1.4	5.0 ± 1.2	0.028
PVR, dyn-s-cm^−5^	202 (155 to 254)	205 (138 to 289)	0.628

Values are given as means ± SD or median and interquartile range or total numbers and percent. AF = atrial fibrillation; 6MWD = 6 min walk distance; NYHA = New York Heart Association; COPD = chronic obstructive pulmonary disease; ACE = angiotensin-converting enzyme; AT II = angiotensin II; HMG-CoA = 3-hydroxy-3-methyl-glutaryl-coenzyme A; NTproBNP = N-terminal prohormone of brain natriuretic peptide; eGFR = estimated glomerular filtration rate; LA = left atrial; LV = left ventricular; RA = right atrial; RV = right ventricular; MOLLI-ECV = modified Look–Locker inversion recovery sequence derived extracellular volume; PAP = pulmonary arterial pressure; PAWP = pulmonary artery wedge pressure; TPG = transpulmonary pressure gradient; PVR = pulmonary vascular resistance; CO = cardiac output. Bold *p*-values indicate statistically significant differences.

**Table 2:** Right atrial volumetric and strain-related parameters.

Variable	Sinus Rhythm (n = 96)	AF (n = 92)	*p*-Value
**Right Atrial Cardiac Magnetic Resonance Imaging Parameters**			
** RA Volumetric Analyses**			
Vi max., mL/m^2^	46 ± 15	85 ± 35	**<0.001**
Vi pre A-wave, mL/m^2^	40 ± 14	/	
Vi min., mL/m^2^	27 ± 11	77 ± 34	**<0.001**
Conduit EF, %	13 ± 9	/	
Booster pump EF, %	28 ± 9	/	
Total EF, %	41 ± 10	11 ± 10	**<0.001**
**RA Strain Analyses**			
Reservoir strain, %	28 ± 12	/	
Conduit strain, -%	15 ± 9	/	
Booster strain, -%	13 ± 5	/	
Reservoir strain rate, %/s	134 ± 63	/	
Conduit strain rate, -%/s	115 ± 67	/	
Booster strain rate, -%/s	148 ± 54	/	

AF = atrial fibrillation; RA = right atrial; Vi = volume index; EF = emptying fraction. Bold *p*-values indicate statistically significant differences.

**Table 3:** Univariable cox regression analysis regarding the association of right atrial parameters to cardiovascular outcome in sinus rhythm and atrial fibrillation.

			Univariate	
Variable	No Event	Event	Hazard Ratio (95% CI)	*p*-Value
** Right atrial magnetic resonance imaging parameters in sinus rhythm**				
	**(n = 62)**	**(n = 34)**		
** RA Volumetric Analyses**				
Maximum Vi, mL/m^2^	47 ± 16	44 ± 14	0.99 (0.97–1.02)	0.662
Vi pre A-wave, mL/m^2^	40 ± 14	39 ± 13	1.00 (0.98–1.03)	0.887
Minimum Vi, mL/m^2^	28 ± 11	26 ± 12	1.00 (0.97–1.03)	0.998
Conduit EF, %	14 ± 10	11 ± 7	0.97 (0.94–1.01)	0.174
Booster EF, %	28 ± 9	30 ± 9	1.02 (0.98–1.06)	0.310
Total EF, %	41 ± 10	41 ± 11	0.99 (0.96–1.03)	0.707
** RA Strain Analyses**				
Reservoir strain, %	30 ± 12	24 ± 11	**0.96 (0.93–0.99)**	**0.034**
Conduit strain, -%	16 ± 9	12 ± 8	**0.94 (0.89–0.99)**	**0.023**
Booster strain, -%	13 ± 5	13 ± 6	0.98 (0.92–1.05)	0.549
Reservoir strain rate,%/s	143 ± 64	117 ± 58	**0.99 (0.98–0.99)**	**0.049**
Conduit strain rate, -%/s	127 ± 71	94 ± 53	**0.99 (0.98–0.99)**	**0.014**
Booster strain rate, -%/s	155 ± 53	134 ± 54	0.99 (0.99–1.00)	0.063
** Adjusted analysis for age, NT-proBNP level, RV EF and NYHA class**				
Reservoir strain			0.95 (0.91–0.99)	0.016
Reservoir strain rate			0.99 (0.98–0.99)	0.028
Conduit strain			0.93 (0.88–0.99)	0.019
Conduit strain rate			0.99 (0.98–0.99)	0.011
** Right atrial magnetic resonance imaging parameters in atrial fibrillation**				
	**(n = 41)**	**(n = 51)**		
** RA Volumetric Analyses**				
Maximum Vi, mL/m^2^	88 ± 32	83 ± 37	0.99 (0.99–1.01)	0.516
Minimum Vi, mL/m^2^	78 ± 33	76 ± 35	0.99 (0.99–1.01)	0.862
Total EF, %	15 ± 13	10 ± 8	0.97 (0.94–0.99)	0.026

CI = confidence interval; RA = right atrial; Vi = volume index; EF = emptying fraction. Bold *p*-values indicate statistically significant differences.

**Table 4:** Correlations of right atrial function and markers of exercise capacity and pulmonary vascular function.

	6MWD m	NT-proBNP ng/mL	sPAP mmHg	PVR dyn-s-cm^−5^
	***ρ***	***ρ***	***ρ***	***ρ***
**Sinus Rhythm**				
**RA conduit function**				
Conduit EF, %	0.26 *	−0.19	−0.20	−0.36 **
Conduit strain, -%	0.18	−0.39 **	−0.32 **	−0.28 *
Conduit SR, -%/s	0.24 *	−0.38 **	−0.29 **	−0.32 **
**RA booster pump function**				
Booster pump EF,%	−0.17	−0.14	0.12	0.07
Booster strain, -%	−0.19	−0.17	0.02	−0.16
Booster SR, -%/s	−0.56	−0.19	0.02	−0.15
**RA total EF, %**	0.07	−0.26 *	−0.10	−0.27 *
**RA reservoir function**				
Reservoir strain, %	0.05	−0.37 **	−0.23 *	−0.31 **
Reservoir SR, %/s	0.04	−0.43 **	−0.08	−0.19
**RA volumetric measurements**				
RAVi endsyst., mL/m^2^	0.05	0.26 *	0.13	−0.05
RAVi pre A, mL/m^2^	−0.01	0.27 **	0.18	0.02
RAVi enddiast.mL/m^2^	0.01	0.32 **	0.13	0.04
**Atrial Fibrillation**				
RA total EF, %	0.13	−0.20	−0.17	−0.18
RAVi endsyst., mL/m^2^	0.07	0.03	−0.01	−0.13
RAVi enddiast.mL/m^2^	0.04	0.09	0.09	−0.10

6MWD = 6 min walk distance; NT-proBNP, N-terminal prohormone of brain natriuretic peptide; sPAP, systolic pulmonary artery pressure; PVR, pulmonary vascular resistance; *ρ* = Spearman correlation; RA = right atrial; EF = emptying fraction; SR = strain rate; Vi = volume index. Bold p-values indicate statistically significant differences. * *p*-value < 0.05; ** *p*-value < 0.01

## Data Availability

The data presented in this study are available on request from the corresponding author.
